# Participatory health research and promotion with children in Germany: a scoping review

**DOI:** 10.1186/s12961-026-01544-8

**Published:** 2026-10-01

**Authors:** Janna Landwehr, Hanna Luetke Lanfer, Miriam Schierenbeck, Leonie M. Heim-Scheffel

**Affiliations:** School of Public Health, Bielefeld University, Universitaetsstrasse 25, 33615 Bielefeld, Germany

**Keywords:** Participatory health research, Participatory health promotion, Children, Adolescent, Peer research, Collaboration, Health equity, Reflexivity, Scoping review

## Abstract

**Background:**

Participatory health research and promotion (PHR/P) emphasizes collaborative knowledge production, mutual learning, and redistribution of power between academic and non-academic actors. With children, PHR/P has gained increasing legitimacy under the United Nations Convention on the Rights of the Child, which recognizes children as rights-holders and social actors. However, little is known about how PHR/P with children is framed and enacted in Germany. This review provides the first systematic overview of PHR/P with children in Germany.

**Methods:**

We conducted a scoping review following Arksey and O’Malley’s framework and PRISMA guidelines. Searches were performed in four databases in March–April 2024, supplemented by snowballing of friends' studies. Eligible studies (2000–2024) reported empirical projects in Germany (plus two projects from German-speaking Switzerland) that explicitly applied participatory approaches with children (< 18 years) and addressed health-related topics. Medical intervention studies and studies limited to clinical patient groups were excluded. Data extraction used a structured codebook adapted from the International Collaboration for Participatory Health Research and focusing on objectives, actors, research phases, and reflections. Two reviewers coded each report independently.

**Results:**

Twelve projects (23 publications) met the inclusion criteria. Most projects were situated in educational or communal contexts. Health-related objectives were consistently reported, often focusing on behavioural change. Participatory objectives were less frequent, remained implicit in several cases, and were rarely defined with children. Children typically participated alongside other adults, rarely as the sole group of non-academic actors. Participation was usually reported in operational phases (e.g. data collection), whereas involvement in conceptual phases (e.g. defining research questions, analysis, dissemination) was uncommon. Creative methods (e.g. photovoice, theater, drawing) were widely used. Reflections focused mainly on ethics, informed consent, and child protection, while sustainability, power relations, and children’s perceived benefits were seldom addressed. Reporting practices were often vague, limiting systematic assessment.

**Discussion:**

PHR/P with children in Germany has gained traction but remains unevenly reported. Health-related goals dominate participatory aims, children’s roles are often mediated by adults, and reflections on sustainability and power remain underdeveloped. Greater transparency in reporting participatory processes is essential to strengthen cumulative knowledge and design meaningful child-centred approaches.

*Trial registration* N/A.

**Supplementary Information:**

The online version contains supplementary material available at https://doi.org/10.1186/s12961-026-01544-8.

## Introduction

Children have increasingly become a focus of participatory research across disciplines, including education, child protection, and health promotion [1–3]. This development reflects a broader paradigm shift in modern societies, in which children are no longer conceptualized solely as objects of concern or protection but as social actors and subjects with their own agency and perspectives [4, 5]. In line with the United Nations Convention on the Rights of the Child [6], which enshrines the right of children to the enjoyment of the highest attainable standard of health (article 24.1), to freedom to seek, receive, and impart information and ideas (article 13.1), and to express their views freely on matters affecting them and to have those views given due weight (article 12.1), participatory research with children has gained legitimacy as both a methodological and ethical imperative [7, 8]. In this review, we use the term children in a broad sense to refer to all individuals under 18 years of age, in line with the UN-CRC [6]. Where relevant, we distinguish between younger children (approximately 6–9 years) and adolescents (children aged 10 years and above [9]). Despite, or perhaps precisely because of the broad use of the term "children", it should be noted that, first, children constitute a group of people that is heterogeneous in many aspects (e.g. development, education, life circumstances). Second, understandings of childhood and their implications are socially constructed [4]. Within the new sociology of childhood, it is argued that, whilst there is not a lack of interest in research **on** children, children should be seen as social actors in their own lives, with those around them, and in the societies in which they live [4].

Participatory approaches **with** children are a promising pathway for addressing health-related issues in ways sensitive to children’s lived realities. However, this does not merely mean that research should be child-centred or geared toward specific needs, rather, a network should be developed in which children and adults share responsibility for the process [3]. They aim to empower children to contribute actively to research and intervention processes, thereby fostering improved health outcomes and children’s capacity for agency and self-determination [10, 11]. This aligns with Freire’s [12] argument that lasting change is most likely achieved through continuous dialogue in which participants become aware of how their own beliefs and behaviours shape their environment. Yet, despite a growing number of studies, questions remain about the extent and nature of children’s participation across different research phases, and about the ways in which power asymmetries (e.g. due to generational differences, adultism), ethical dilemmas (e.g. care and self-determination), and researcher reflexivity are or should be addressed [13, 14]. These tensions and dilemmas have spurred debates about how participatory health research and health promotion with children can be meaningfully designed while respecting ethical boundaries and acknowledging differences among participants [3, 14].

Moreover, the sociopolitical context in which such participatory projects take place must be considered. Germany, for instance, is shaped by distinctive institutional arrangements in welfare, health care, and education, which influence how children’s participation is framed and practiced [15, 16]. Although children’s rights have been politically prominent for years, they are not yet explicitly enshrined in the German constitution [17], leaving important questions open about how participation is enacted in practice. Compared with other countries (e.g. the USA, UK, Canada [11]), the development of participatory approaches with children has only recently gained renewed momentum, and the number of published studies remains limited [18, 19]. Moreover, Germany’s demographic development, including increasing social diversity, raises additional challenges and opportunities for inclusive participatory approaches. By focusing on Germany, this review examines how participatory health research and promotion with children is currently understood and enacted in this specific sociopolitical and institutional context. This review is part of a broader project that examines participatory health research and promotion with "seldom-heard" groups and applies the same analytical framework across populations to enable systematic comparison. It has already been applied in a parallel review on PHR/P with migrants [20].

## Conceptual background

Participatory research approaches refer to collaborative approaches in which generally academic and non-academic actors jointly generate knowledge and develop strategies for health improvement. Rather than treating participants as passive subjects, they emphasize shared ownership, mutual learning, and the redistribution of power within research processes [21, 22].

The field of participatory approaches is diverse; within it, participatory health research and participatory health promotion represent closely related yet distinct strands that share a common commitment to democratizing knowledge production and practice. Participatory health research (PHR) is typically defined as a collaborative scientific approach in which academic and non-academic actors work together throughout the research process to generate actionable knowledge and foster social change [23]. Participatory health promotion (PHP), in turn, emphasizes co-producing interventions and strategies to improve health and well-being, often at the community or organizational level [24]. For this review, we adopt an inclusive perspective on both domains, referring to them as participatory health research and promotion (PHR/P).

PHR/Ps are known to come with tensions and dilemmas, including the risk of tokenism, the challenges of sustaining collaboration under resource constraints, and the difficulty of balancing protection with empowerment [21, 22, 25]. When children are involved, these challenges intersect with additional complexities arising from generational asymmetries, legal responsibilities, and societal expectations about children’s roles in research [3, 8]. This makes it essential to situate participation with children within broader theoretical debates.

PHR/Ps with children thus require careful attention to epistemological and normative dimensions. Epistemologically, research with children challenges adult-centred assumptions by showing that children’s views are not incomplete versions of adult knowledge but valuable insights into their own lived realities [5, 26]. Normatively, because children are recognized as rights-holders under international frameworks such as the United Nations Convention on the Rights of the Child [6], participation should also enable them to meaningfully influence decisions that concern them [27, 28]. Together, these considerations highlight the need to examine how participation with children is actually practiced and described in published studies. To do so, this review focuses on four key aspects.

The first aspect concerns the objectives that PHR/P with children set out to achieve. On the one hand, the recognition of children as social actors has underlined the importance of designing projects that build on children’s own perspectives, priorities, and lived experiences [5, 26]. On the other hand, research agendas in health contexts are often strongly shaped by adult professionals, academic frameworks, and institutional priorities, which may sideline or redefine children’s concerns [10, 29]. Scholars have noted that this creates a persistent tension between child-centred knowledge production and adult-defined goals, as well as between the logic of health promotion and the logic of academic research [30]. While individual studies have highlighted these challenges, little systematic evidence remains on how objectives are framed in published projects. Our review, therefore, addressed the following question:

### RQ1

What are the stated health- and participatory-related objectives in PHR/P with children in Germany?

The second aspect concerns the actors who are described as participants in PHR/P. While the concept of participation suggests children as co-researchers, the literature shows that studies frequently involve a broader set of actors, including parents, teachers, social workers, and peer groups [16, 31]. Throughout this review, we use "actors" as an umbrella term for all academic and non-academic participants who take part in projects alongside the children as co-researchers. Including these additional actors may support children’s engagement but can also blur participation boundaries, raising questions about inclusivity, representativeness, and adult gatekeeping [32–34]. Scholars have highlighted these dilemmas, but we lack a consolidated overview of who is formally described as participating in published work. Against this background, our review asks:

### RQ2

Who is described as participating in PHR/P with children in Germany?

The third aspect concerns the phases of the research process in which participation occurs. In its principles, participatory research aims to engage co-researchers in all phases, from identifying research questions and designing methodologies to collecting data, analysing findings, and disseminating results [27, 28]. However, authors have also noted that participation may not always be possible or desirable because of differences in resource availability (e.g. time, finances, capabilities) or interest in the topic [35, 36]. This generates a tension between the ideal of participation as a comprehensive principle and its reality as a selective contribution [37, 38]. Continuous reflection has therefore been emphasized as a way to ensure that children’s opportunities for decision-making and informed consent are realized as fully as possible [36]. As there is limited systematic evidence on how far children are reported to be involved across the research cycle, our review asks:

### RQ3

In which research phases are children described to participate in PHR/P in Germany?

The fourth aspect concerns critical reflections on ethics, power, and sustainability in PHR/P with children. Researchers must navigate dilemmas of informed consent, confidentiality, and unequal power relations while balancing the imperative to protect children with the commitment to recognize their agency [8, 33, 35, 36]. Moreover, frequently asked ethical questions remain: whether participation produces lasting changes in children’s lives [10], whether these changes are seen as beneficial by the children themselves, or whether the children's own benefits are reflected [8, 10, 39]. Hence, we ask:

### RQ4

Which project-related reflections are described in publications about PHR/P with children in Germany?

## Methods

This scoping review followed the methodological framework of Arksey and O’Malley [40], and it is reported in accordance with PRISMA guidelines [41]. We chose a scoping review because our aim was to map the breadth of an emerging and heterogeneous field of research and to provide a comprehensive overview of how PHR/P is conducted with children, a purpose for which scoping reviews are well suited. The review builds directly on our previous scoping review of PHR/P with migrant communities in Germany [20]. In that earlier work, we developed a coding framework for analyzing participatory processes, which was iteratively refined and pilot-tested before being applied to the full set of included studies. In the present review, we applied the same framework and analytical procedure to ensure comparability across population groups.

### Search strategy and study selection

The literature search was conducted in four databases (PubMed, LIVIVO, Web of Science, Psyndex) and guided by four categories: participatory research, children, health, and Germany. The categories and their synonyms were linked between each by the Boolean operators and/or as shown in Table 1. The following search string was used for PubMed and adapted accordingly to ensure compatibility with the indexing and search functionalities of the other databases (see Supplementary Material for the other search strings). Both English and German search terms were used; German terms were applied in particular for the German-language databases (LIVIVO and Psyndex). Where available, we used MeSH terms, which also retrieve related indexed concepts. The literature search was conducted in March and April 2024, separately for each database.

**Table 1:** Overview of search categories and terms

Category	Search terms
Participation	Participatory[Title/Abstract] OR “participatory health research”[Title/Abstract] OR “participatory research”[Title/Abstract] OR CBPR[Title/Abstract] OR participation[Title/Abstract] OR “community based participatory research”[Title/Abstract] OR Teilhabe[Title/Abstract] OR collaborat*[Title/Abstract] OR community based participatory research[MeSH Terms]
Population	Adolescent[MeSH Terms] OR child[MeSH Terms] OR child development[MeSH Terms] OR adolescent development[MeSH Terms] OR schoolage[Title/Abstract] OR kid[Title/Abstract] OR boy[Title/Abstract] OR girl[Title/Abstract] OR youngster[Title/Abstract] OR child*[Title/Abstract] OR adolescen*[Title/Abstract] OR teenage[Title/Abstract]
Health	Health Promotion[MeSH Terms] OR Public Health[MeSH Terms] OR prevention[Title/Abstract] OR “primary prevention”[Title/Abstract] OR health[Title/Abstract] OR School Health Services[MeSH Terms]
Country	Germany[MeSH Terms] OR german*[Title/Abstract]

*Literature search in PubMed, 22 March 2024, 1174 records

In addition, we invited further publications via the PartNet mailing list, which serves as a central network for participatory health research in Germany and hosts a dedicated working group on participatory research with children, to identify relevant publications not retrieved from the databases. We also applied a snowballing procedure (“friends studies”) to capture additional relevant publications that were directly assessed against the inclusion criteria.

All references were collated manually in Word and PDF lists; duplicates were removed by hand. Titles and abstracts were screened against the eligibility criteria (see Table 2) by two independent reviewers. In case of disagreement, a third person was consulted. Potentially relevant articles were subsequently assessed in full text, with disagreements resolved through discussion. Where multiple publications described the same project, they were grouped and analysed together at the project level to avoid duplication.

**Table 2:** Inclusion and exclusion criteria for PHR/P with children in Germany

Criteria	Inclusion	Exclusion
Language	English or German	Publications in other languages
Publication date	From 2000 onwards	Published before 2000
Geographical focus	Studies conducted in Germany or focused on children in Germany	Studies outside Germany or lacking explicit focus on Germany
Focus population	Involvement of children	No involvement of children
Research approach	Author-reported participatory research (e.g. PHR, CBPR, community-based) or participatory health promotion	Non-participatory studies
Health focus	Health-related studies	Non-health-related studies; medical studies (e.g. medical intervention studies, focus on patients with diagnosed underlying conditions)
Publication type	Qualitative, quantitative, or mixed-methods studies	Theoretical articles without an underlying study, review articles, commentaries, presentations, reviews, etc.
	Report of one or more empirical studies (authors describe their own study)	
Document availability	Full texts accessible via databases, institutional access or mailing list (PartNet); articles without listing could be included as friends studies	Abstracts only or inaccessible texts

**Fig. 1:**
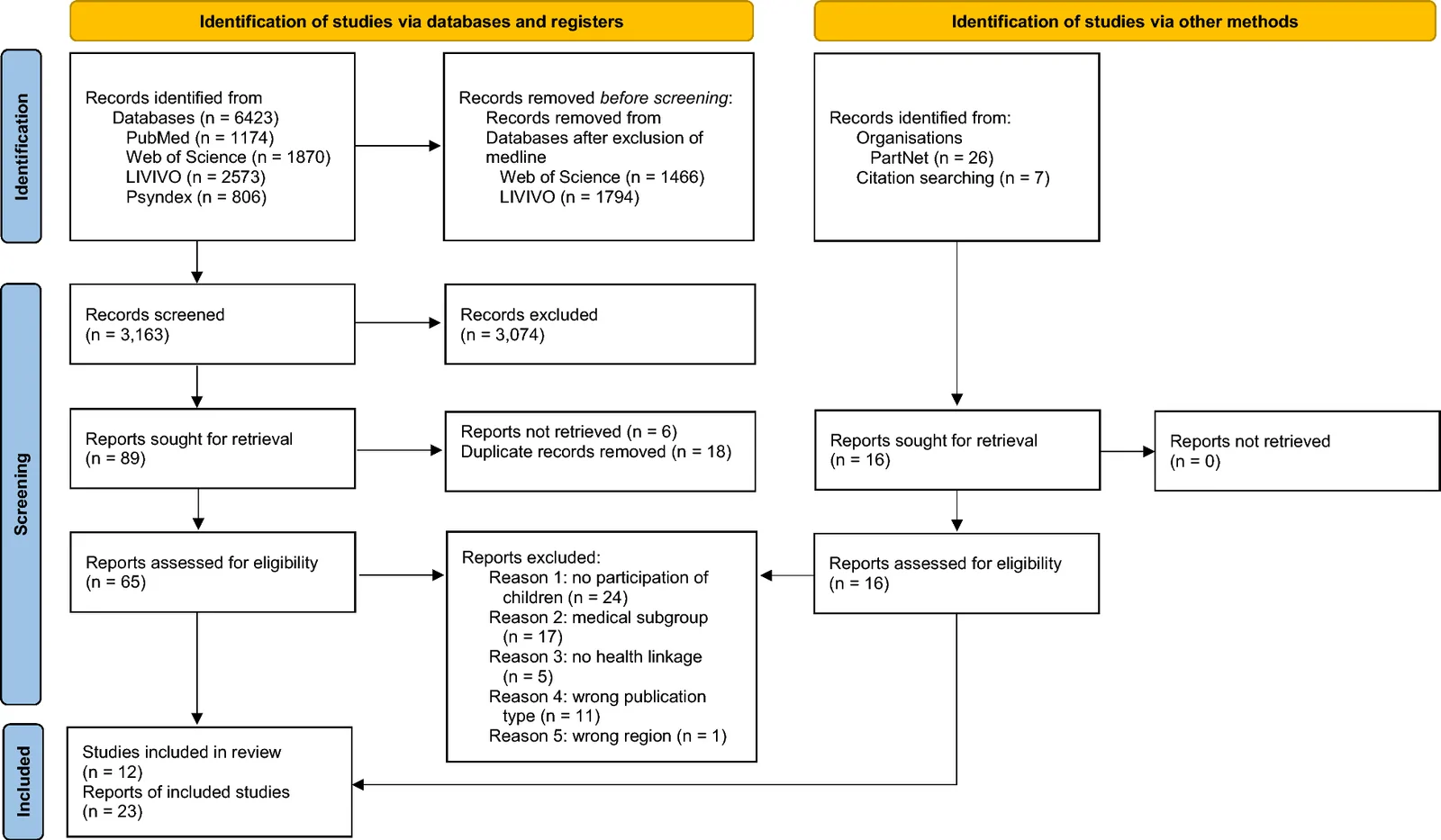
PRISMA flowchart. *Source* Page MJ, et al. BMJ 2021;372:n71. 10.1136/bmj.n71. For more information, visit: https://www.prisma-statement.org/

As outlined in Table 2, we applied the following eligibility criteria: A study was included if it used a participatory approach and explicitly stated that it worked participatively with children as the focus population. Projects with a health-related focus were considered. In contrast, medical studies such as medical intervention studies or studies focused on patients with diagnosed underlying conditions (e.g. intervention studies with children with physical disabilities, neurological disorders, oncology patients, or mental illness) were excluded. Furthermore, German- and English-language publications from Germany were eligible for inclusion. Although our review focused on Germany, we also included two projects from the German-speaking part of Switzerland, as they are embedded in German research networks. The time frame for inclusion was set from the year 2000 onwards. Only qualitative, quantitative, or mixed-methods studies that reported one or more empirical studies conducted by the authors themselves were included, while other publication types were not eligible. Additionally, all articles had to be listed in scientific databases. The process is documented in the PRISMA flowchart (Fig. 1).

### Data extraction and coding

Data extraction was guided by a structured codebook derived from the International Collaboration for Participatory Health Research “Characteristics of Participatory Health Research” [38] and refined through iterative piloting in our earlier review. Categories covered: (1) publication characteristics, (2) theoretical conceptualization and terminology, (3) methodological approach, (4) participation across research phases, and (5) reflections on participation.

The coding team consisted of four members: three student assistants and the first author. The first author wrote two of the included publications. To minimize bias, title/abstract and full-text screening for these records were conducted independently by two other reviewers. Coding for these publications was likewise performed by two other team members; the first author neither coded nor reconciled coding for her own publications. Discrepancies for these records were adjudicated by one of the two coders. While the first author checked overall coding consistency across the dataset, she did not take part in inclusion decisions or discrepancy resolution for her own publications.

To ensure consistency, all coders participated in a test-coding phase, independently coding two publications, resulting in three codings per publication. Discrepancies were discussed. Inter-rater reliability was calculated using ReCal_3_ [42], yielding an overall Cohen’s kappa of 0.81, indicating strong agreement.

For the main analysis, two reviewers (a student assistant and the first author) coded each included full text independently, and they reconciled diverging results through discussion. For projects represented by multiple publications, coding was aggregated at the project level.

### Analytical procedure

All coding was conducted using Excel. RQs 1–4 were addressed based on the closed categories of the codebook. These categories were coded numerically (1 = Yes, 2 = Not named, 3 = No), allowing systematic comparisons across projects and providing a structured overview of participatory practices. In addition, we used open categories to capture further nuances beyond the extracted numerical data, e.g. if "Other" or “Unclear” was coded. For these categories, relevant text passages were extracted into the Excel cells and linked back to the original PDFs to ensure traceability. Moreover, each reviewer reflected on the coding process at different points in time. The team shared and discussed these reflections.

As in our earlier review, we did not conduct a formal appraisal of the study quality. Our aim was to examine the processes of participation within participatory projects, hence how participation is conceptualized and enacted, rather than to assess methodological rigour or health-related outcomes.

## Results

The included publications covered 12 distinct studies/projects^1^ with 23 reports, most of which were classified as projects predominantly in health promotion and three as studies in health research (nos. 5, 7, 9). Most of the projects were implemented in educational and/or communal settings. Only a few projects were situated outside these contexts (e.g. no. 8 in broader urban settings).

Target groups included school-going children and adolescents, with several studies covering broad age ranges (e.g. nos. 9, 12), with only one project including both pre-school and primary-school-going children during an observational period (no. 2). Regarding thematic orientation, a strong emphasis was placed on physical health (nos. 1, 2, 3, 8, 9, 10, 11, 12) [e.g. physical activity (e.g. nos. 3, 9), healthy nutrition (e.g. no. 1)]. Projects addressing environmental health (no. 4) and mental health (nos. 5, 6, 7) were comparatively rare [e.g. cyberbullying prevention (no. 5), strengthening mental health (no. 6)]. Two of the included projects addressed other topics such as health literacy (no. 4) and open space development (no. 12). All but one project (no. 1) were coded as mixed methods (see Table 3).

**Table 3:** Characteristics of included projects

No.	Author, year	Study design	Methods used with children/youth	Author identified type of research	Health-related objectives	Participatory-related objectives	Study setting	Focus study population	Other stakeholder recruited	Authorship	Type of publication
1	Fischer et al. [43]	Quantitative	Interactive method: Vegetation surveys [inventory of plant species, species abundance and herbaceous species, classification (native, edible)]	Participatory health promotion	(i) Improved food and environmental education, (ii) increased availability of locally grown, fresh, and seasonal produce for pupils, and (iii) enhanced awareness, use, and management of local urban biodiversity	Collaborative activities in planning, managing, and using the school garden and wild wasteland site	School, neighbouring surrounding	Children and adolescents (10–18 y)	School staff, local government representatives, local environmental organization, researchers, moderation team from the field of urban green infrastructure planning	Academic researchers Stakeholders from non-academic institutions	Peer-reviewed article
2	(a) Igel et al. [44] (b) Igel et al. [45]	Mixed methods	Interactive method: Group work for intervention development (exploring daily routine, work-out types, and facilitators of active locomotion)	Participatory health promotion	Preventing childhood obesity and increasing healthy behaviours in children living in a deprived district	Developing strategies, representation in project planning processes	Community area, schools	Children (4–12 y) observed, (6–9 y) participated	Local stakeholders (government, institutions, associations, residents), landscape architect, intervention test sample	Academic researchers	Peer-reviewed articles
3	(a) Landwehr and Kolip [46] (b) Landwehr and Kolip [47]	Mixed methods	Interactive, creative method: Photovoice (taking pictures, group discussions, development of recommendations, dissemination)	Participatory health promotion	Promoting physical activity in terms of active transport	Assessing (1a) practicability of the method (1b) needs of children	Community area, school	Children (6–9 y)	Not named	Academic researchers	Peer-reviewed articles
4	Maitz and Gasteiger-Klicpera [48]	Mixed methods	Interactive method: workshops (input, individual and group work, plenary discussions)	Participatory health promotion	Strengthening health literacy	Not named	School	Adolescents (12–15 y)	Medical students	Academic researchers	Peer-reviewed article
5	(a) Mameli et al. [49] (b) Hamilton et al. [50] (c) Purdy et al. [51]	Mixed methods	Interactive, art-based method: Quality circle (group sessions: analysing an issue, transfer into comics, presentation)	Participatory health research and promotion	Increasing awareness and health literacy of cyberbullying	(7a) Understanding the phenomenon from an insider perspective; increasing usability and effectiveness of resources (7b) Empowering young people and strengthening cooperation and problem-solving competences (7c) not named	School	Adolescents (14–16 y)	School administration, moderators	Academic researchers	(a, b) Peer-reviewed articles (c) Contribution to anthology
6	Pfister et al. [52]	Mixed methods	Interactive, creative method: brainstorming, photovoice (taking pictures, group discussions, development of recommendations or intervention, dissemination), lightning survey	Participatory health promotion	Strengthening mental health	Contributing local knowledge	Community area, school	Adolescents (13–18 y)	Public health professionals, graphic and text designer	Academic researchers, stakeholders from non-academic institutions	Peer-reviewed article
7	Rataj et al. [53]	Mixed methods	Survey, interactive method: workshop (reflection on survey results)	Participatory health research	Strengthening autonomy to promote mental health and resilience in youth	Contributing interests and strengthening competences	Community area	Adolescents (16–18 y)	Professionals of youth work	Academic researchers	Peer-reviewed article
8	Riegger [54]	Mixed methods	Interactive creative method: Brainstorming, model drawing/construction	Participatory health promotion	Promoting movement-friendly urban development to prevent developmental disorders, addiction, traffic hazards and lack of physical activity	Focusing on needs of girls and women for salutogenetic urban development	Community area	Children and adolescents (9–16 y)	Professionals of youth welfare and public construction office	Academic researchers	Contribution to anthology
9	(a) Scheller et al. 2024 [55] (b) Scheller and Bachner [56]	Mixed methods	Interactive, creative method: Photovoice with walking interviews and mapping exercises (taking pictures, conducting interviews and GPS-tracking during a walk, group discussion)	Participatory health research	Promoting physical activity	Strengthening underrepresented participants by having an active role in research	Community area	Children and adolescents (6–17 y)	University students of sports or health sciences	Academic researchers	Peer-reviewed articles
10	Stölzel et al. [57]	Mixed methods	Interactive method: group discussion with planning group; survey (written questionnaire, ranking)	Participatory health promotion	Strengthening health literacy and prevention of skin cancer	Including most affected people in planning process	School	Children and adolescents (10–18 y)	Teachers, coaches, school administration	Academic researchers	Peer-reviewed article
11	(a) Strobl et al. [58] (b) Ptack et al. 2020 [59] (c) Sauter and Loss [60] (d) Gelius et al. [61]	Mixed methods	Interactive method: group discussions within planning groups	Participatory health promotion	Strengthening health-related knowledge and understanding for physical education	Strengthening capacity-building processes	School	Children and adolescents (10–18 y)	Physical education teachers and school administration	Academic researchers	(a, c, d) Peer-reviewed articles (b) Contribution to anthology
12	(a) Widmer and Stutz [62] (b) Fabian 2016 [63] (c) Fabian and Käser [64] (d) Fabian and Huber [65]	Mixed methods	Interactive, creative method: pin method, subjective maps, walk-through, auto-photography, exploration and assessment, planning-drawing workshop, model construction, participatory building days	Participatory health promotion	Designing open, child-friendly spaces for health promotion in urban areas	Participating in development and decision-making processes, documenting the "voice", sensitizing for democracy, planning and implementation processes, capacity-building	Community area, school	Children and adolescents (6–18 y, focus 6–12 y)	Municipality stakeholders, experts for planning, architecture and participation, school	Academic researchers	(a) Final report of external evaluation (b) Peer-reviewed article (c) Non-peer-reviewed article (d) Contribution to anthology

### Stated health- and participatory-related objectives in PHR/P with children (RQ1)

All included projects reported health-related objectives, most often linked to the project topic, e.g. promoting physical activity (e.g. no. 9) and raising awareness of sun protection (no. 10). Most projects targeted behavioural objectives of the child or their family (nos. 4, 5, 6, 7, 9, 10, 11), whereas social or structural objectives (nos. 1, 2, 3, 8, 12), e.g. creating supportive environments, with active transport infrastructures (no. 2) or green spaces (no. 1) were less common (see Table 3).

Participatory objectives, by contrast, were articulated much less consistently than health-related objectives. In a few cases, they were stated only briefly or remained implicit (e.g. nos. 4, 6). Where explicitly formulated, they covered a range of objectives, including strengthening children’s role in research (no. 9) or exploring the needs of children (no. 3). Objectives were generally not defined together with the children. Only one project reported that they were at least discussed and could be adapted (no. 6).

### Actors described as participating in PHR/P with children (RQ2)

All projects included minors and academic researchers as participants, yet they rarely constituted the only group involved (see Table 3). In all but one project (no. 3), participation took place in multiactor constellations that often included teachers and school staff alongside children (nos. 1, 4, 5, 9, 10, 11). Some studies also involved welfare professionals or community representatives (nos. 1, 2, 6, 7, 8, 12), showing the high prevalence of adult participants in PHR/P.

### Phases of participation in PHR/P with children (RQ3)

The reviewed publications reported children’s involvement across different phases of the research process, but participation was usually described in concrete phases such as data collection (all included projects) or idea generation (e.g. nos. 5, 12; see Table 4). Fewer projects reported children’s participation in conceptual phases (nos. 3, 5, 6, 7) such as refining and adapting research questions, analyzing data, or contributing to dissemination. Furthermore, no publication shared authorship with the children even though children were thanked in the acknowledgements of half of the projects (nos. 1, 2, 5, 6, 7, 9, 10).

**Table 4:** Author-identified participation of focus population in different research phases

No.	Author,year	Main/focus study population	Conceptualization of research project (including defining research question and objective)	Operationalisation of research instruments (including feedback to surveys, pilot testing, and translations)	Data collection	Data analysis	Publications, policy papers, other outputs	Workshops, conferences for distribution of results	Other
1	Fischer et al. [43]	Children and adolescents (10–18 y)	Not named	Not named	Yes	Not named	Not named	Not named	Usage of garden and kitchen, results discussion in urban learning laboratory with other local projects
2	(a) Igel et al. [44] (b) Igel et al. [45]	Children (4–12 y) observed, (6–9 y) participated	Not named	Yes	Yes	Not named	Not named	Not named	–
3	(a) Landwehr and Kolip [46] (b) Landwehr and Kolip [47]	Children (6–9 y)	No	No	Yes	Yes	No	No	–
4	Maitz and Gasteiger-Klicpera [48]	Adolescents (12–15 y)	No	Not named	Yes	Not named	Not named	Not named	Co-creation of workshops to develop a computer-assisted tool for health literacy strengthening
5	(a) Mameli et al. [49] (b) Hamilton et al. [50] (c) Purdy et al. [51]	Adolescents (14–16 y)	No	Not named	Yes	Not named	Yes	Not named	Creation of comics and further materials to illustrate cyberbullying
6	Pfister et al.[52]	Adolescents (13–18 y)	Yes	Yes	Yes	Yes	Not named	Yes	–
7	Rataj et al. [53]	Adolescents (16–18 y)	No	Yes	Yes	Yes	Not named	Yes	–
8	Riegger [54]	Children and adolescents (9–16 y)	No	Not named	Yes	Not named	Not named	Not named	–
9	(a) Scheller et al. 2024 [55] (b) Scheller and Bachner [56]	Children and adolescents (6–17 y)	Not named	Not named	Yes	Not named	Not named	Not named	–
10	Stölzel et al. [57]	Children and adolescents (10–18 y)	Not named	Yes	Yes	Not named	Not named	Not named	Co-creation of video spots to strengthen health literacy
11	(a) Strobl et al. [58] (b) Ptack et al. 2020 [59] (c) Sauter and Loss[60] (d) Gelius et al.[61]	Children and adolescents (10–18 y)	No	Not named	Yes	Not named	Not named	Not named	Development of sport lessons according to student-centred teaching strategies for implementation and discussion of experiences
12	(a) Widmer and Stutz [62] (b) Fabian 2016 [63] (c) Fabian and Käser [64] (d) Fabian and Huber [65]	Children and adolescents (6–18 y, focus 6–12 y)	Not named	Yes	Yes	Not named	Not named	Not named	Analysis of the initial situation; plan design and concept; Realization/implementation

In terms of methodology, it stood out that many projects reported using interactive methods like workshops or trainings (all projects but no. 10). Moreover, several used creative, arts-based approaches (nos. 2, 3, 5, 6, 8, 9, 12), including photovoice, drawing, or theater exercises.

Notably, reporting practices varied. In many cases, the descriptions of when and how children participated during different phases remained vague, leading to a high number of unclear codings. Fewer studies gave detailed accounts of the phases and the respective roles of children and other actors (nos. 3, 5, 6, 7). Vagueness was also found in relation to the participation of other actors, making it difficult to determine to which extent other actors participated and whether children were the main participants (e.g. no. 1). Overall, reported participation by different participants across the research cycle was selective and often insufficiently specified.

### Reflections on participation in PHR/P with children (RQ4)

Reflections on the participatory process were reported in all but one project (no. 8), although the level of detail varied (see Table 5). Ethical aspects were addressed frequently (nos. 2, 3, 4, 5, 7, 9, 11), particularly questions of informed consent and balancing children’s protection with their agency (e.g. nos. 3, 5). The availability and distribution of resources (nos. 1, 4, 5, 9, 10, 11, 12), including challenges of maintaining motivation (e.g. nos. 6, 12) and methodological reflections about the tools and methods used, were also common (nos. 3, 4, 5, 6, 9, 10, 11, 12).

**Table 5:** Author-identified project-related reflections

No.	Author,year	Main/focusstudy population	Challenges of/withinparticipatory activities	Ethicalaspects	Methods (recruitment, data collection, etc.)	Availability and distribution of resources of different stakeholders	Influence of (external) events	Positionality and role of academic researchers
1	Fischer et al. [43]	Children and adolescents (10–18 y)	Yes	Not named	Not named	Yes	Not named	Not named
2	(a) Igel et al. [44] (b) Igel et al. [45]	Children (4–12 y) observed, (6–9 y) participated	Not named	Yes	Not named	Not named	Yes	Not named
3	(a) Landwehr and Kolip [46] (b) Landwehr and Kolip [47]	Children (6–9 y)	Not named	Yes	Yes	Not named	Yes	Not named
4	Maitz and Gasteiger-Klicpera [48]	Adolescents (12–15 y)	Not named	Yes	Yes	Yes	Not named	Not named
5	(a) Mameli et al. [49] (b) Hamilton et al. [50] (c) Purdy et al. [51]	Adolescents (14–16 y)	Yes	Yes	Yes	Yes	Not named	Yes
6	Pfister et al. [52]	Adolescents (13–18 y)	Yes	Not named	Yes	Not named	Yes	Not named
7	Rataj et al. [53]	Adolescents (16–18 y)	Not named	Yes	Not named	Not named	Yes	Yes
8	Riegger [54]	Children and adolescents (9–16 y)	Not named	Not named	Not named	Not named	Not named	Not named
9	(a) Scheller et al. 2024 [55] (b) Scheller and Bachner [56]	Children and adolescents (6–17 y)	Yes	Yes	Yes	Yes	Yes	Not named
10	Stölzel et al. [57]	Children and adolescents (10–18 y)	Yes	Not named	Yes	Yes	Not named	Not named
11	(a) Strobl et al. [58] (b) Ptack et al. 2020 [59] (c) Sauter and Loss [60] (d) Gelius et al. [61]	Children and adolescents (10–18 y)	Yes	Yes	Yes	Yes	Yes	Not named
12	(a) Widmer and Stutz [62] (b) Fabian 2016 [63] (c) Fabian and Käser [64] (d) Fabian and Huber [65]	Children and adolescents (6–18 y, focus 6–12 y)	Yes	Not named	Yes	Yes	Yes	Not named

Most reflections on impact remained short and descriptive. Sustainability and longer-term impact were mentioned only sporadically (e.g. no. 5). Similarly, critical self-reflection varied across projects: while some publications offered more nuanced insights into dilemmas or tensions in their participatory approach (nos. 1, 5, 6, 9, 10, 11, 12), others focused primarily on documenting activities. In contrast to RQ3, which often required unclear coding due to vague reporting, the reflections were more clearly coded, with most entries falling into yes or *not named*.

## Discussion

This scoping review assessed how carrying out participatory health research and promotion (PHR/P) with children in Germany has been described since 2000. Across the 12 included projects, several overarching observations emerge that align with international debates while also showing gaps in reporting about PHR/P with children.

### Summary and discussion of study findings

Our finding that participatory objectives (RQ1) were often only briefly stated or remained implicit resonates with broader debates in the literature. Interestingly, this vagueness persisted even in cases where projects were represented by multiple publications, up to four in some instances, and with occasionally identical passages (e.g. no. 12), suggesting that a higher number of outputs does not necessarily translate into more detailed reporting of participatory processes. Scholars have long noted that while participation is widely invoked, it is rarely operationalized as a goal in its own right [1, 66]. Instead, health-related objectives, often behaviour-oriented, tend to dominate, whereas participatory or empowerment-oriented objectives are less frequently in the foreground [11, 67]. This pattern reflects the persistent tension between acknowledging children as social actors [4] and framing their participation primarily in relation to health outcomes.

Our results indicate that children rarely appear as the only participants in PHR/P (RQ2) and are often accompanied by school staff or other adult stakeholders. This mirrors insights from childhood studies as children’s agency is often mediated through generational and institutional structures [3, 16]. It also raises questions about how adult decision-makers frame children and their agency in participatory projects. Hence, while multiactor constellations may enhance ecological validity and facilitate implementation in schools or communities, they also risk diluting children’s voices. The literature has emphasized this dilemma, asking whose perspectives are ultimately prioritized in participatory projects [14, 15]. Our review adds to this as reporting practices often remained vague in this regard, making it difficult to assess how influence was distributed between children and adults.

Children were most frequently reported to participate in operational project phases (RQ3), such as data collection or idea generation, while involvement in conceptual phases like defining research questions or analyzing data was much rarer. This is consistent with earlier observations that children’s input is welcomed but seldom extends to shaping research agendas or analytical frameworks [7, 10]. This pattern extended to dissemination, as no publication reported children as co-authors or clarified whether and how results were communicated back to them. It is therefore questionable whether children who had contributed to other phases of the research were positioned to access or use the findings themselves, or what other personal benefit they derived from the research. At the same time, the prominence of creative, arts-based methods, such as photovoice, drawings, or theater, stood out. It marks a departure from traditional reliance on surveys or structured interviews for data collection and may be viewed as a way to make PHR/P engaging and accessible [10, 68]. On the other hand, their labelling as "child-friendly" may also suggest an adultistic construction of childhood and deficit-oriented assumptions about what children can and cannot do (e.g. low on concentration, memory). However, as our review shows, reporting often lacked clarity about whether these methods enabled children to exercise genuine influence over research decisions.

Regarding reflexivity (RQ4), ethical considerations, particularly informed consent, the balance between protection and agency and building trust, were among the most frequently reported reflections, echoing long-standing debates in participatory research [3, 8, 36]. Methodological reflections, especially on the appropriateness of creative tools, were also common, suggesting that standards in this area are becoming more established. In contrast, structural dimensions such as sustainability and long-term impact, as well as reflections on power (of adults and children or power relations) and researchers' positionality, received much less attention. Few publications considered whether children themselves experienced participation as beneficial. Yet previous work has underscored that these aspects are critical to avoiding tokenism and ensuring participation beyond the life of a single project, and it points to unevenness in reported reflections [10, 69]. This unevenness may also connect to structural barriers within academic systems of third-party funding, which prioritize short-term projects and measurable outcomes over long-term participatory engagement, and raises the question of how research designs can realistically foster continuity and impact under such institutional constraints.

Building on these findings, we suggest that future publications on PHR/P with children move beyond stating that children participated toward specifying the phases in which they were involved, including analysis and dissemination, and the degree of decision-making influence they held at each. Reporting explicitly whether and how children participated in each phase would make visible how far participation extended. The codebook used in this review, which distinguishes between these phases, offers researchers and practitioners a concrete starting point for documenting these dimensions in future PHR/P with children.

### Comparison of applying the same coding framework to two reviews

By conducting two scoping reviews with the same analytical framework, one on PHR/P with migrants and one with children, we were able to identify some overarching patterns that go beyond the specific study populations. In both reviews, publications were our sole data source, meaning we did not observe the participatory practice itself, but rather how it was presented and constructed in the academic literature. Across both reviews, a striking commonality lies in the vagueness with which participatory processes were often described. Participatory-related objectives, phases of involvement, and reflections were often addressed in broad or unspecific terms, leaving ambiguity about what participation actually entailed in practice. In the children’s review, this vagueness was even more pronounced: theoretical grounding, explicit reflection, and actual involvement across phases of the research process were reported in less depth than in the migrant review. While such vagueness may partly reflect the constraints of publication formats—where process reflections are often seen as secondary to reporting results, constrained by space limitations and a demand for "success stories"—it limits other scholars' ability to learn systematically from prior experiences. Findings from both reviews underscore the importance of creating spaces where dilemmas, constraints, and methodological choices can be reported with greater clarity. If such accounts were more consistently integrated into publications, they would allow for stronger cumulative knowledge about how PHR/P can be meaningfully adapted to diverse contexts and population groups.

### Strengths and limitations

This review has several strengths. First, to our knowledge, it is the first systematic overview of PHR/P with children in Germany, thereby providing a baseline for future scholarship and practice. Second, developing and applying a structured codebook to two different subpopulations allows for direct comparison of PHR/P across subfields. While a detailed synthesis of the results of both reviews is beyond the scope of this article, the shared codebook provides the basis for such comparative analyses and may be applied to other subpopulations. Finally, we provide a methodological tool for systematically assessing how participatory processes are reported across different populations and contexts. This codebook, consisting of structured questions, may also be of use to researchers aiming to reflect on or document participation in their own projects.

Some limitations must be acknowledged. We deliberately did not assess study quality or health outcomes, as our focus was on participation processes rather than intervention effects. Our findings are restricted to what was reported in publications and therefore cannot fully capture how participation was enacted in practice. Moreover, several projects were represented in multiple publications and, therefore, we could capture more detail during coding than with single-publication projects. The literature search reflects the evidence available up to April 2024; studies published thereafter fall outside the defined search period. Finally, we included only projects recorded in scientific databases that excluded reports and other published accounts of PHR/P with children. It is therefore likely that additional studies exist which were not captured because they focused on children with specific health conditions (and were excluded due to their medical framing), because the journals in which they were published were not indexed in the databases searched, or because authors described their work with terms other than "participation", such as "peer research". This assumption echoes Clar and Wright’s [70] finding that participatory projects were often not published as peer-reviewed, scientific articles.

## Conclusion

To our knowledge, this scoping review provides the first systematic overview of participatory health research and promotion with children in Germany. Guided by four research questions, we analyzed how objectives, actors, phases of participation, and reflections were reported across 12 projects. Taken together, the review shows that while participatory approaches with children have increased over the years, reporting on the extent of participation often remains vague and uneven. By using a structured codebook, this study not only offers insights into the field's current state but also provides a tool for assessing and comparing how participatory processes are documented. Strengthening transparency in reporting will be crucial for building cumulative knowledge and for supporting researchers and practitioners in designing participatory approaches that are meaningful to both children as co-researchers and academic researchers.

## Supplementary Information

Additional file 1.

## Data Availability

The datasets used during the current study are available from the corresponding author on reasonable request.

## Abbreviations

ICPHR: International Collaboration for Participatory Health Research
PHR/P: Participatory health research and promotion
RQ: Research question
UN-CRC: United Nations Convention on the Rights of the Child

## Footnotes

1 Due to the combination of PHR and PHP, we will use the term "projects" in the following as an umbrella term for studies and projects.
