# Supplementary material for: Participatory health research and promotion with children in Germany: a scoping review
Source: Health Res Policy Syst. 2026 Oct 1;24:84. doi: 10.1186/s12961-026-01544-8 (PMC13632410; doi:10.1186/s12961-026-01544-8)
Supplement: Supplementary file 1 [file 12961_2026_1544_MOESM1_ESM.docx]

**Search strings**

| Database | PubMed | LIVIVO | Web of Science | Psyndex |
| --- | --- | --- | --- | --- |
| Search string | (((((((((((participatory[Title/Abstract]) OR ("participatory health research"[Title/Abstract])) OR ("participatory research"[Title/Abstract])) OR (CBPR[Title/Abstract])) OR (participation[Title/Abstract])) OR ("community based participatory research"[Title/Abstract])) OR (Teilhabe[Title/Abstract])) OR (collaborat*[Title/Abstract])) OR (community based participatory research[MeSH Terms])) AND ((Adolescent[MeSH Terms]) OR (((((((((((child[MeSH Terms]) OR (child development[MeSH Terms])) OR (adolescent development[MeSH Terms])) OR (schoolage[Title/Abstract])) OR (kid[Title/Abstract])) OR (boy[Title/Abstract])) OR (girl[Title/Abstract])) OR (youngster[Title/Abstract])) OR (child*[Title/Abstract])) OR (adolescen*[Title/Abstract])) OR (teenage[Title/Abstract])))) AND ((Germany[MeSH Terms]) OR (german*[Title/Abstract]))) AND (((((((Health Promotion[MeSH Terms]) OR (Public Health[MeSH Terms])) OR (prevention[Title/Abstract])) OR ("primary prevention"[Title/Abstract])) OR (health[Title/Abstract])) OR (health promotion[MeSH Terms])) OR (School Health Services[MeSH Terms])) | **(participatory OR "participatory health research" OR "participatory research" OR CBPR OR participation OR "community based participatory research" OR Teilhabe OR collaborat*) AND (adolescent OR child OR schoolage OR kid OR boy OR girl OR youngster OR Kind OR Jugendlich OR teenage) AND (Germany OR german* OR Deutsch*) AND (“Health Promotion" OR “Public Health” OR prevention OR "primary prevention" OR health OR Gesundheit OR Prävention*)**  *(excluded databases: Medline, Psyndex)* | (((TS=(participatory OR "participatory health research" OR "participatory research" OR CBPR OR participation OR "community based participatory research" OR collaborat*)) AND TS=(Adolescent OR child OR schoolage OR kid OR boy OR girl OR youngster OR child* OR adolescen* OR teenage)) AND TS=(Germany OR german*)) AND TS=(“Health Promotion” OR “Public Health” OR prevention OR "primary prevention" OR health OR “health promotion”)  *(excluded database: Medline)* | All text Fields (participatory or participatory health research or participatory research or CBPR or participation or community based participatory research or collaborat* or Teilhabe or Partizipation) AND (adolescents or child or schoolage or kid or boy or girl or youngster or teenage or kind or Jugendlich) AND (germany or german or deutschland or deutsch) AND (health promotion or public health or prevention or primary prevention or health or Gesundheit or Prävention) |
| Date | 22.03.2025 | 22.03.2024 | 10.04.2024 | 16.04.2024 |
| Records | **1,174** | **779** | **404** | **806** |
|  |  |  |  |  |

**Coding Book Review Participatory health research and promotion with ‘seldom-heard’ groups [20]**

Note: The codebook also entails categories for coding that were not reported in the review.

| **Category** | **Subcategories** | **Guiding questions** | **Characteristics** |
| --- | --- | --- | --- |
| **General description** | Title of the study | What is the title of the study? | Please describe, including DOI |
|  | Authorship | Who are named as authors? | 1 = Academic researchers  2 = Stakeholders from non-academic instituons  3 = Authors without instituional affiliation  4 = Co-researchers/Peer researchers  5 = Other |
|  |  | If 2 - 5, please specify | Please describe and mark in the text |
|  | Type of publication | What type of publication is it? | 1 = Book chapter  2 = Article in collected work/compilation  3 = Article in peer reviewed journal  4 = Article in a non peer-reviewed journal |
|  | Year of Publication | In which year was the study published? | Please describe and mark in the text |
|  | Topic | What is the health-related topic of the project? | Please describe and mark in the text |
|  | Type of project | What type of participatory approach does the publication report about? | 1 = Participatory health promotion  2 = Participatory health research |
|  | Funding | Was the study or project sponsored by a third party? | 1 = Yes  2 = Not named  3 = No |
|  |  | If yes (1), which one? | Please name paragraph, programme or institution |
| **Study population** | Included groups | Which study population is the focus of the project? | 1 = People with a migration background/migrant communities  2 = Children |
|  |  | If 1, which countries of origin do the persons come from? | Name countries of origin and exact migration background (if described) |
|  |  | If 2, what age group of children are involved?  Please specify (multiple answers possible): | 1 = Infants (0-5)  2 = Young children (6-9)  3 = Older children (10-12)  4 = Young teenagers (13-15)  5 = Older teenagers (16-18) |
|  |  | Are there other socio-demographic categories in the focus study population that are explicitly taken into account? | 1 = Yes  2 = Not named  3 = No |
|  |  | If yes (1), which one (s)? | 1 = Gender  2 = Age  3 = SES  4 = 1st generation of immigrants  5 = 2nd generation of immigrants  6 = legal status (e.g. refugees, asylum seekers)  6 = other (name) |
|  |  | If Gender (1), which one? | Please describe and mark in the text |
|  | Excluded groups | Are certain groups explicitly excluded according to the description? | 1 = Yes  2 = Not named  3 = No |
| **Introduction of participation as a concept and terminology** |  | If yes (1), which one and for what reason? | Please describe and mark in the text |
|  | Recruitment | How is the participating study population in focus recruited (snowball method, doctors, etc.)? | 1 = Stakeholder  2 = Institutions  3 = Snowball method  4 = Peers/ people from the community  5 = Other  6 = Not named |
|  |  | If peers/people from the community (4), please name/describe and mark in the text. |  |
|  | Theoretical consideration  of participation | Do the authors utilise theoretical models, concepts, frameworks for participation (e.g. stage model of participation, participatory health research model)? | 1 = Yes  2 = Not named  3 = No |
|  |  | If yes (1), which one? | Please describe and mark in the text |
|  |  | Are further theoretical models/theories introduced and explained (e.g. health competency, health literacy)? | 1 = Yes  2 = Not named  3 = No |
|  |  | If yes (1), which one? | Please describe and mark in the text |
|  | Terminology | What terms do the authors use to describe the participatory study (multiple answers possible)? | 1 = Participatory health research (PHR)  2 = Community-based participatory research (CBPR)/Community-based participatory research  3 = Action Research  4 = Participatory health promotion  5 = Collaborative research  6 = Health services research  7 = Other  8 = None |
|  |  | If Other (7), which? | Please describe and mark in the text |
|  |  | How are the respective terms defined/introduced? | Please describe and mark in the text |
|  |  | How are the people involved who are not described as being academic researchers(multiple answers possible)? | 1 = Real world researchers  2 = Co-researchers  3 = Researchers from experience/Experts by experience  4 = Research team member  5 = Participants  6 = Peer researchers  7 = Other |
|  |  | If Other (7), which one? | Please describe |
| **Objectives and their effects** | Health-related objectives | Which health-related objectives does the project pursue (e.g. promoting physical activity, improving mental health, health literacy) (multiple answers possible)? | Please describe |
|  | Participatory-related objectives | What are the overarching objectives of the study? (multiple answers possible) | 1 = Development of an intervention  2 = Exploration/generation of knowledge about challenges & opportunities of a health-related topic  3 = Development of a survey/research instrument (e.g. questionnaire)  4 = Behavioural change (e.g. increase in exercise in everyday life);  5 = Collect and strengthen resources (e.g. social networks, financial/material resources)  6 = Other  7 = Not named |
|  |  | If Other (6), please specify. |  |
|  |  | Is there explicit description of the objectives related to participation (e.g. empowerment, representation, redistribution of power)? | 1 = Yes  2 = Not named  3 = No |
|  |  | If Yes (1), what are the objectives for the participants? | Please describe and mark in the text |
|  | Effects | To what extent were these objectives defined with all groups/stakeholders? | 1= All goals were defined together  2 = Participatory researchers each have defined sub-goals  3 = Goals were discussed and could be processed/adjusted  4 = Goals were justified  5 = Targets have been set  6 = Not named  7 = Other |
|  |  | If No information (6) or Other (7), please specify. |  |
|  |  | Is the achievement of the participatory objectives reported? | 1 = Yes  2 = Not named  3 = No |
|  |  | If Yes (1), how is the achievement of the participatory-related objectives reported? | Please describe and mark in the text |
|  |  | What impact do the authors describe in relation to the participatory objectives (e.g. degree of participation, networking, improvement of equal opportunities)? | Please describe and mark in the text |
|  |  | Are the interests/expected benefits of various stakeholders reported? | 1 = Yes  2 = Not named  3 = No |
|  |  | If Yes (1), please name and mark in the text. |  |
| **Methodological approach** | Study design | Which study design was used? | 1 = Cross-sectional study  2 = Long-term/multiple  surveys/pretest-posttest  3 = With control group  4 = Without control group  5 = Unclear  6 = other |
|  |  | If Unclear (5) or other (6), please describe and mark in the text. |  |
|  | Duration of study | In which period did the whole study take place? | Please name (in months) |
|  |  | In which period did data collection take place? | Please name (in weeks) |
|  | Intervention | Was there an intervention related to the health goal (e.g. sports course to promote physical activity, nutrition course for weight reduction)? | 1 = Yes  2 = Not named  3 = No |
|  |  | If Yes (1), please describe and mark in the text. |  |
|  |  | If several study populations participated in the study - who participated in the intervention? | Please describe and mark in the text |
|  |  | In which setting did the intervention take place? | 1 = Municipality/ community centre  2 = place of learning (school, university of applied sciences, community college)  3 = Workstation/workplace  4 = long-term residence (e.g. hospital, residential group, other forms of accommodation...)  5 = Other |
|  |  | If Other (5), please name and mark in the text. |  |
|  | Further measures | Have other activities been carried out to prepare or promote the project (e.g. training of participatory community staff/participants, information event on the study, relationship building through additional meetings)? | 1 = Yes  2 = Not named  3 = No |
|  |  | If Yes (1), please name and mark in the text. |  |
|  | Data collection | Which method(s) were used in the data collection (multiple answers possible)? | 1 = Written questionnaire/survey  2 = Interviews  3 = Focus groups  4 = Experiment  5 = Ethnographic methods  6 = creative methods (e.g. drawings, photos, theatre etc.)  7 = Mix of methods  8 = other |
|  |  | If Creative methods, mix of methods or other (6-8), which ones? | Please describe and mark in the text |
|  |  | If several study populations are participating in the study - who took part in which data collection? | Please describe and mark in the text |
|  |  | What types of knowledge were collected? | 1 = professional expertise  2 = academic/scientific knowledge  3 = Patient knowledge  4 = Network knowledge  5 = social and life-world knowledge  6 = non-biomedical, nonevidence-based health/specialist knowledge  7 = Other |
|  |  | If Other (7), please describe and mark in the text. |  |
|  |  | How was the data of the focus study population collected (multiple answers possible)? | 1 = independently completed  2 = collected through interaction with another person (face-to-face, by telephone)  3 = Other |
|  |  | If Other (3), please describe and mark in the text. |  |
|  |  | Who collected the data? (multiple answers possible) | 1 = academic/scientific researchers  2 = lifeworld/ peer/co-researchers  3 = Other |
|  |  | If Other (3), please describe and mark in the text. |  |
|  |  | In which setting did the data collection take place? | 1 = Municipality/ community centre  2 = place of learning (school, university of applied sciences, community college)  3 = Workstation/workplace  4 = long-term residence (e.g. hospital, residential group, other forms of accommodation...)  5 = Other |
|  |  | If Other (5), please name and mark in the text. |  |
|  | Linguistic materials | Were the communication materials (consent form, data collection, etc.) adapted to the focus study population? | 1 = Yes  2 = Not named  3 = No |
|  |  | To what extent were the communication materials (consent form, data collection, etc.) adapted to the focus study population? | 1 = Difficulty level/comprehensibility (e.g. easy language)  2 = written translation into the participants' native language  3 = Visualisations  4 = other persons present (e.g. translator, integration worker, etc.)  5 = Other  6 = Not or not named |
|  |  | If 1 - 5, please describe and mark in the text. |  |
|  | Data analysis | What methods were used to analyse the data? | 1 = Thematic/ content analysis  2 = Grounded Theory  3 = Statistical methods  4 = Other |
|  |  | If Other (4), please describe and mark in the text. |  |
| **Research process** | Participation in phases of the research project | Do the authors differentiate between different types of involvement/participation in the study design? | 1 = Yes  2 = Not named  3 = No |
|  |  | If Yes (1), how are they described and defined? Please Describe and mark in the text. |  |
|  |  | Are people in the key study population part of steering or decision-making bodies (e.g. advisory board, steering group, etc.)? | 1 = Yes  2 = Not named  3 = No |
|  |  | If Yes (1), how is this defined? Please describe and mark in the text. |  |
|  |  | In which phases is the focus study population actively/participatively involved (e.g. other peers interviewed, involved in data analysis) (multiple answers possible)? | 1 = Recruitment of participants  2 = Creation of survey instruments  3 = Data collection  4 = Data analysis  5 = Publications, policy papers, other outputs  6 = Workshops, conferences to discuss/distribute the results  7 = Other (please specify) |
|  |  | For 1 - 7 please specify for each type of participation and mark in the text. |  |
|  |  | In which phases does the degree of participation of the focus study population remain unclear (e.g. through passive constructions/descriptions)? | 1 = Recruitment of participants  2 = Creation of survey instruments  3 = Data collection  4 = Data analysis  5 = Publications, policy papers, other outputs  6 = Workshops, conferences to discuss/distribute the results  7 = Other (please specify)  8= None |
|  |  | From which phases of the research process was the focus study population explicitly excluded? | 1 = Recruitment of participants  2 = Creation of survey instruments  3 = Data collection  4 = Data analysis  5 = Publications, policy papers, other outputs  6 = Workshops, conferences to discuss/distribute the results  7 = Other (please specify)  8 = None |
|  |  | If there were explicit exclusions, on what grounds? | Please specify and mark in the text |
|  |  | In which phases are other study populations actively/participatively involved (multiple answers possible)? | 1 = Recruitment of participants  2 = Creation of survey instruments  3 = Data collection  4 = Data analysis  5 = Publications, policy papers, other outputs  6 = Workshops, conferences to discuss/distribute the results  7 = Other (please specify)  8 = None |
|  | Output and processing of the results | Apart from this scientific publication being coded, are other outputs/processing of the results mentioned? | 1 = Yes  2 = Not named  3 = No |
|  |  | If Yes (1), please specify and mark in the text. |  |
| **Reflection** | Effect | Are the effects of the participatory study design reflected? | 1 = Yes  2 = Not named  3 = No |
|  |  | If Yes (1), please describe and mark in the text. |  |
|  |  | Were there any unintended effects (positive and negative)? | 1 = Yes  2 = Not named  3 = No |
|  |  | If Yes (1), please describe and mark in the text. |  |
|  | Ethics | Were concepts for the protection of participants (e.g. ethics application, data protection concept) developed in advance of the study? | 1 = Yes  2 = Not named  3 = No |
|  |  | If Yes (1), please describe and mark in the text. |  |
|  |  | Were the concepts for protecting the participants critically reflected upon in the course of the study? | 1 = Yes  2 = Not named  3 = No |
|  |  | If Yes (1), please describe and mark in the text. |  |
|  | Methodology | Is the methodological approach reflected in the context of participation? | 1 = Yes  2 = Not named  3 = No |
|  |  | If Yes (1), please describe and mark in the text. |  |
|  | Context | Are (differences in) living conditions/life circumstances of all participants reflected? | 1 = Yes  2 = Not named  3 = No |
|  |  | If Yes (1), please name and mark in the text. |  |
|  |  | Are the assumptions/attributions of the academic/scientific researchers reflected? | 1 = Yes  2 = Not named  3 = No |
|  |  | If Yes (1), please name and mark in the text. |  |
|  |  | Is the influence of available resources (including experience) of the various participants reflected? | 1 = Yes  2 = Not named  3 = No |
|  |  | If Yes (1), please name and mark in the text. |  |
|  |  | Is the influence of (external) events (e.g. pandemic, influence of external persons, departure of participants/key positions, loss of resources) reflected? | 1 = Yes  2 = Not named  3 = No |
|  |  | If Yes (1), please name and mark in the text. |  |
|  | Role of researchers in the research process | Do the academic researchers reflect on their role (role conflicts) with regard to interactions (resources, power, hierarchies, education, time) with the other participants (e.g. through a positioning/positionality statement)? | 1 = Yes  2 = Not named  3 = No |
|  |  | If Yes (1), please specify and mark in the text. |  |
|  |  | Does the study address leadership, management and decision-making authority? | 1 = Yes  2 = Not named  3 = No |
|  |  | If Yes (1), please specify and mark in the text. |  |
|  | Work process | Is there reflection on the creation of a shared working atmosphere (building trust, conflict resolution, listening, joint learning...)? | 1 = Yes  2 = Not named  3 = No |
|  |  | If Yes (1), please specify and mark in the text. |  |
|  |  | Is it reported if financial Compensation was made for co-researchers? | 1 = Yes  2 = Not named  3 = No |
|  |  | If Yes (1), please specify and mark in the text. |  |
|  |  | Is there reflection on the development of the group/the group feeling for participants (e.g. empathy, sense of togetherness, appreciation)? | 1 = Yes  2 = Not named  3 = No |
|  |  | If Yes (1), please name and mark in the text. |  |
|  | Challenges and how to deal with them | Are challenges with participation reported? | 1 = Yes  2 = Not named  3 = No |
|  |  | If Yes (1), please name and mark in the text. |  |
|  |  | Are unpredictability, chaos and the fact that these are part of the participatory research process reflected upon? | 1 = Yes  2 = Not named  3 = No |
|  |  | If Yes (1), please specify and mark in the text. |  |
|  |  | Are lessons learnt/recommendations given for participatory projects? | 1 = Yes  2 = Not named  3 = No |
|  |  | If Yes (1), please name and mark in the text. |  |
|  | Literature references | Is further literature by the authors on the aforementioned project or other own writings related to participation listed? | 1 = Yes  2 = Not named  3 = No |
|  |  | If Yes (1), please name and mark in the text. |  |
| **Comments and notes from the coders** |  |  |  |
